# Comparison of McGrath^®^ Series 5 video laryngoscope with Macintosh laryngoscope: A prospective, randomised trial in patients with normal airways

**DOI:** 10.12669/pjms.324.10037

**Published:** 2016

**Authors:** Mehmet Sargin, Mehmet Selcuk Uluer

**Affiliations:** 1 Mehmet Sargin, MD. Anesthesiology and Reanimation Department, Konya Training and Research Hospital, Konya, Turkey; 2 Mehmet Selcuk Uluer, MD. Anesthesiology and Reanimation Department, Konya Training and Research Hospital, Konya, Turkey

**Keywords:** Airways, Laryngoscopes, Tracheal intubation, Video laryngoscopes

## Abstract

**Objective:**

The McGrath Video laryngoscope is a newly developed video laryngoscope that significantly improves laryngeal view and facilitates endotracheal intubation in difficult airways. However in patients with normal airway that is not mentioned before. The aim of this study was to compare the McGrath video laryngoscope with the Macintosh laryngoscope in patients with normal airways.

**Methods:**

A total of 100 patients requiring orotracheal intubation, were randomized to either having intubation with the McGrath video laryngoscope or the Macintosh laryngoscope. The primary outcome was the laryngoscopy view using percentage of glottic opening (POGO) score. Secondary outcomes included Cormack and Lehane grading system, time to intubation, number of failed intubations, number of attempts before successful intubation and hemodynamic parameters during intubation.

**Results:**

POGO scores were significantly higher in the McGrath group compared with the Macintosh group (p<0.001) despite time to successful intubation was similar in both groups. The McGrath video laryngoscope provided more Grade-I laryngoscopic views than the Macintosh laryngoscope (p<0.001). Number of more than one attempts in order to achieve success was significantly higher in the Macintosh group (p=0.001). The number of minor complications were significantly higher in the Macintosh group (p=0.004). There were no significant changes in hemodynamic responses between the two groups (p>0.05).

**Conclusion:**

McGrath video laryngoscope allows patients with normal airways to achieve higher POGO scores and significantly more Grade-I laryngoscopic views when compared with the Macintosh laryngoscope.

## INTRODUCTION

Tracheal intubation is an important step in securing airway patency during general anesthesia. Hence, rapid, flawless, successful intubation process is of primary importance for anesthesiologists. Macintosh direct laryngoscope is the most commonly used laryngoscope in the intubation of the patients. In the presence of difficulty at intubation, complications such as dental injury, laryngospasm, bronchospasm, and severe complications such as hypoxia, hypercarbia, and death can occur as a result of multiple intubation attempts.^1^

Therefore, many alternative devices have been developed to facilitate smooth intubation, and the utility and benefits of these devices have been compared in various studies. Video laryngoscopes are at the top of the list of devices developed for the purpose of smooth intubation. This device has many types and the number of such devices is increasing every passing day.

We studied one such alternative intubation device: the McGrath^®^ Series 5 video laryngoscope (Aircraft Medical Ltd, Edinburgh, UK). The McGrath video laryngoscope is designed to provide a better laryngeal view than that obtained by direct laryngoscopy with a Macintosh laryngoscope. It also provides that with a high-resolution video camera placed within an angulated single-use blade of adjustable length. Several studies have reported improved laryngoscopic views and greater intubation success rates in patients with predicted and known difficult airways using the McGrath video laryngoscope.^2^-^7^ However, there are no prospective and randomized clinical trials comparing the McGrath video laryngoscope with the Macintosh laryngoscope in patients with normal airways. The primary outcome was the laryngoscopic view using percentage of glottic opening (POGO) score with both devices. Secondary outcomes included Cormack and Lehane grading system, time to intubation, number of failed intubations, number of attempts before successful intubation, complications and hemodynamic parameters during intubation.

## METHODS

After obtaining approval from local Institutional Review Board (Necmettin Erbakan University Meram School of Medicine Ethics Committee) and registration from the Australian New Zealand Clinical Trials Registry (registration number: ACTRN12614000910606), we recruited 100 patients of ASA physical status 1-2 who were scheduled for elective surgery under general anesthesia requiring tracheal intubation.

Airway difficulty score (ADS) 1 was assessed prior to intubation in order to exclude those predicted to make the procedure difficult. Patients with an ADS score above 8 and thyroid-to-chin length of 5 cm or shorter, a Mallampati class 3 or higher, mouth opening less than 3cm, restriction in neck extension or protruding front teeth were predicted to be difficult in intubation and/or airway and were thus excluded from the study. Also, patients were excluded from the study if they required rapid sequence induction, had a history of previous difficult direct laryngoscopy and had uncontrolled hypertension, ischemic heart disease, acute or recent stroke or myocardial infarction, cervical spine instability or cervical myelopathy, symptomatic asthma or reactive airway disease and history of gastric reflux.

After obtaining written informed consent, patients were assigned, by using a computer-generated block randomisation, to laryngoscopy with either McGrath video laryngoscope or the Macintosh laryngoscope. All tracheal intubations with both the Macintosh laryngoscope and McGrath video laryngoscope were performed by one anesthesiologist who had used both devices more than 50 times clinically.

All patients were expected to fast 6-8 hours before surgery, and no one premedicated. With the patient placed in the supine position, routine monitors (consisting of a pulse oximeter, 3-lead ECG and a non-invasive blood pressure cuff) were applied. Baseline measurements were obtained and three minutes of pre-oxygenation was performed before the induction of general anesthesia. Standardize anesthetic induction was performed with 1 mcg/kg fentanyl, 1-2 mg/kg of propofol, and when consciousness was lost, 0.6 mg/kg of rocuronium was injected. After, making sure that all four TOF responses of the adductor pollicis disappeared, which ensures sufficient muscular blockade, intubation was then performed. Number 3 or 4 blades was used in all patients. A size 7.0 mm tracheal tube was used to intubate the trachea in female patients, and a size 7.5 mm tube was used for all male patients.

If more than one intubation attempt was required, the patient received bag-and mask ventilation between attempts and various maneuvers were employed, including external laryngeal pressure, readjustment of the stylet and use of a bougie. Failed intubation was defined as failure after three attempts and a pre-determined alternative airway management plan was instituted by the treating anesthetist. Correct placement of the tracheal tube was confirmed by capnography and bilateral chest auscultation.

The primary outcome was the laryngoscopic view using percentage of glottic opening (POGO) score (0 to 100%, 100 = full view of glottis from anterior commissure to the inter-arytenoid notch, 0 = even inter-arytenoid notch is not seen). Secondary outcomes included Cormack and Lehane grading system (including 1–4), time to intubation, number of failed intubations, number of attempts before successful intubation and hemodynamic parameters (HR: Heart rate, MBP: Mean blood pressure) during intubation. Minor complications like oropharyngeal trauma or mucosal bleeding were also recorded. These data were collected by one independent observer. The time taken for successful tracheal intubation was measured from the time the allocated laryngoscope was inserted in the patient’s mouth until end-tidal carbondioxide was detected.

To ensure that the sample sizes of the study groups would support a valid comparison, a power analysis was performed (α = 0.05, β = 0.90), which indicated that at least 46 subjects should be recruited for each group. For this calculation, we used the mean (67.6) and SD (24.7) of the POGO score with Macintosh laryngoscope, in that study 8 and assumed a 25% increase of POGO score from the Macintosh laryngoscope POGO score value with McGrath laryngoscope. Therefore, we recruited 100 patients in total to account for possible drop-outs.

Statistical analyses were performed with SPSS 15.0 software (SPSS Institute, Chicago, IL, USA). The Mann–Whitney U-test was used to analyze the time for successful intubation and independent t-test used for POGO scores. The Fisher exact test was used to analyze the Cormack and Lehane view at laryngoscopy, the number of intubation attempts and the number of failed intubations. For variables with multiple measurements (hemodynamic variables), a repeated measures analysis of variance was used to evaluated the effects of time and group assignment, while two-way analysis of variance was used to compare the difference within the groups.

## RESULTS

**Fig.1:**
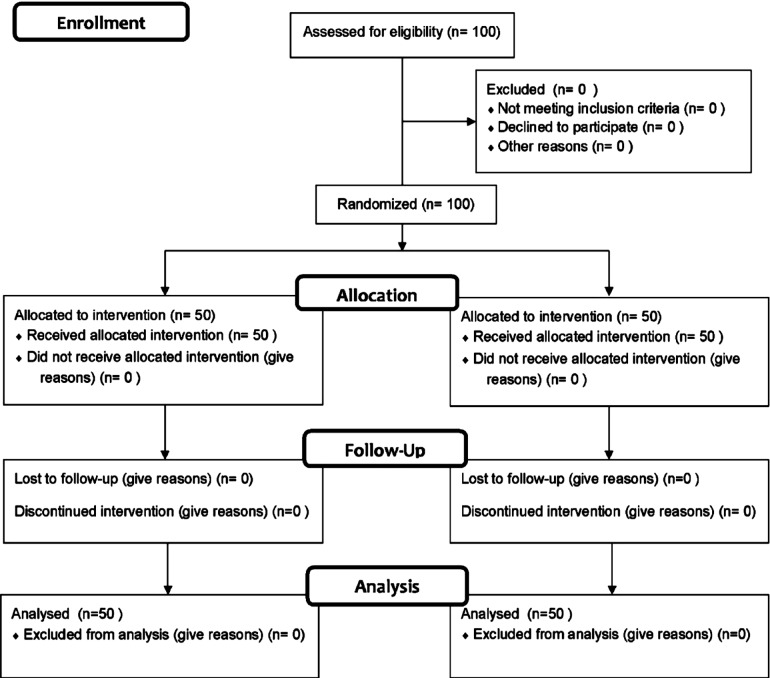
CONSORT flowchart detailing patient recruitment.

The baseline characteristics of the patients were similar in both groups, as shown in Table-I. The median time taken to perform successful intubation was similar in the McGrath group when compared with the Macintosh group (mean 18.93 vs 19.68 s, median 18 vs 18 s, IQR 15-21.5 vs 15-23; Fig.1).

**Table-I:** Comparison of patients’ baseline characteristics between the Mc Grath and Macintosh groups. Values are mean (SD) or number.

	Macintosh Group (n=50)	Mc Grath Group (n=50)	p value
Age (Year)	40.36 (14.67)	39.46 (15.58)	0.771
Gender (Male/Female)	17/33	23/27	0.112
BMI (kg/m^2^)	24.97 (4.13)	24.23 (3.41)	0.346

**Fig.2:**
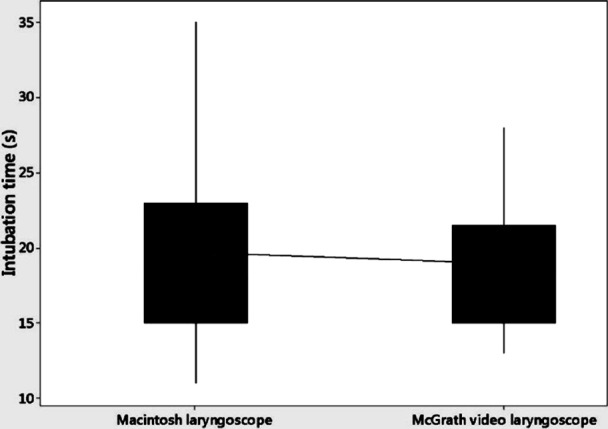
Comparison of time to intubation (s) between the McGrath video laryngoscope group and the Macintosh laryngoscope group. Values are in median, inter-quartile range, maximum and minimum.

The results of the Cormack and Lehane grade, percentage of glottic opening during laryngoscopy, number of intubation attempts, failed intubations and complications are summarized in Table-II.. The McGrath video laryngoscope provided a significantly better laryngoscopic view compared with the Macintosh laryngoscope. POGO scores were significantly higher in the McGrath group compared with the Macintosh group (p<0.001). The McGrath video laryngoscope provided more Grade-I laryngoscopic views than the Macintosh laryngoscope (p<0.001). Although number of more than one attempts in order to achieve successful intubation was significantly higher in the Macintosh group compared with the McGrath group (p=0.001), there were no failed intubation in both groups. There were in total fifteen minor oropharyngeal mucosal injuries as a result of the intubation, two from using the McGrath video laryngoscope and thirteen from the Macintosh laryngoscope. This difference was statistically significant (p=0.004).

**Table-II:** Comparison of the laryngoscopic view, POGO score, the number of intubation attempts and the number of failed intubations between the Mc Grath and Macintosh groups.

		Macintosh group (n=50)	McGrath group (n=50)	p value
CL laryngoscopic view	1	23	40	<0.001
	≥2	27	10	
POGO score (%)		60.80 (35.49)	84.67 (19.39)	< 0.001
Number of intubation attempts	1	43	50	0.001
	>1	7	0	
Number of failed intubations		0	0	
Minor complications Mucosal bleeding		13	2	0.004

Values are number or mean (SD).CL: Cormack and Lehane, POGO: Percentage of glottic opening

The results of hemodynamic parameters. In both groups, there were no significant changes in heart rate (HR) and mean blood pressure (MBP) from baseline to post intubation (Immediately after intubation and two minute after intubation) (p>0.05) are shown in Table-III. There was no statistically significant difference in the changes between the two groups (p>0.05).

**Table-III:** Comparison of the hemodynamic parameters between the McGrath and Macintosh groups. Values are number or mean (SD).

	Macintosh group (n=50)	McGrath group (n=50)	p value
HR_t0 (beat/min)	83.90 (12.77)	80.35 (13.83)	0.194
HR_t1	90.94 (12.31)	88.48 (13.47)	0.352
HR_t2	84.88 (12.74)	81.35 (13.30)	0.187
MBP_t0 (mmHg)	93.16 (10.95)	91.87 (11.49)	0.575
MBP_t1	101.28 (15.01)	102.98 (18.20)	0.618
MBP_t2	92.88 (14.38)	90.46 (14.96)	0.421

HR: Heart rate, MBP: Mean blood pressure, t0: Basal, t1: Immediately after intubation, t2: 2 minute after intubation.

## DISCUSSION

Compared with the Macintosh laryngoscope, the McGrath video laryngoscope improved the laryngeal view in patients with normal airway. The McGrath video laryngoscope was also superior in terms of intubation attempts and minor complications. Despite all these advantages there was no significant difference in duration of intubation.

The studies that used various video laryngoscopes including the Macintosh laryngoscope and McGrath video laryngoscope on patients with normal airways and on manikins have reported intubation times similar to our study.^5^,^8^-^10^ Consistent with our findings, the studies that compared video laryngoscope with the Macintosh laryngoscope reported higher POGO score and higher Grade I laryngoscopic view in video laryngoscope groups.^8^,^10^ Some studies that evaluated intubation times on manikins have reported different results compared to our study. Among these studies, Kim et al. compared the McGrath video laryngoscope with the Glide Scope Ranger portable video laryngoscope, and they reported a two-fold longer intubation times in the McGrath video laryngoscope group compared to our study.^11^ Different from our results, another study performed on manikins reported longer intubation times in the McGrath video laryngoscope group (median time: 40.7 sec, IQR: 31.0-57.4) compared to the Macintosh laryngoscope group.^12^ In another manikin study Ruetzler et al. compared five video laryngoscopes and conventional direct laryngoscopy on simple and simulated difficult airways on the intubation trainer.^13^ Similarl to our findings they revealed in the normal intubation setting, time to intubation ranged from 16.0s (conventional direct laryngoscopy) to 34.3s (McGrath).

However, it must be kept in mind that intubation times in the Macintosh laryngoscope group in that study (median time: 26.1 sec, IQR: 19.9-33.5) were longer compared to intubation times in our study in the Macintosh laryngoscope group. Higher intubation times reported in these two studies compared to our study are considered to be caused by the fact that these studies were conducted on manikins.

The studies that used the McGrath video laryngoscope in patients with difficult airways revealed an intubation time ranging between 35 and 50 seconds.^2^,^14^,^15^ Among these studies, the study comparing the Macintosh laryngoscope with the McGrath video laryngoscope detected longer intubation times in the McGrath video laryngoscope group.^2^ Likewise, although shorter intubation times were achieved with the Henderson direct laryngoscope compared to the McGrath laryngoscope, the difference was not statistically significant.^15^ These periods are different than those found in the present study due to the differences in the patient population. Although intubation times were longer in the McGrath video laryngoscope group, the rate of acquiring a Cormack and Lehane Grade I laryngoscopic view was significantly higher in the McGrath video laryngoscope group. Parallel to this, the percentage of glottic opening (POGO) score was higher in the McGrath video laryngoscope group compared to the Macintosh laryngoscope group.^2^ The present study found similar results in terms of the POGO score and the rates of the Cormack and Lehane laryngoscopic view.

In a study in which inexperienced anesthesiologists used the McGrath video laryngoscope and the Macintosh laryngoscope, the mean intubation time in the McGrath video laryngoscope group (median time: 47.0 sec, IQR: 39.0-60.0) was significantly higher compared to the Macintosh laryngoscope group (median time: 29.5 sec, IQR: 23.0-36.8).^16^ In spite of this, the number of patients with whom the Cormack and Lehane Grade I laryngoscopic view was obtained, was higher in the McGrath video laryngoscope group. However, the overall rate of successful tracheal intubation was similar in both groups. Similar intubation times were found in a study comparing novice users of the McGrath video laryngoscope with the Macintosh laryngoscope on manikins. However, the rate of the Cormack and Lehane Grade I laryngoscopic view was significantly higher in the McGrath video laryngoscope group.^17^ Furthermore, in this study, novices achieved a higher overall rate of successful tracheal intubation when using the McGrath video laryngoscope. In a study about tracheal intubation with a McGrath® Series 5 video laryngoscope by novice personnel in a cervical-immobilized manikin, similar to our results first-attempt success rate was higher for the McGrath® Series 5 compared to the Macintosh laryngoscope (84.2% vs. 47.7%, respectively).^18^ In this study number of more than one attempts in order to achieve successful intubation was significantly higher in the Macintosh group compared with the McGrath group.

The studies in the literature have found different results in terms of minor complications. Some studies comparing the McGrath video laryngoscope with the Macintosh laryngoscope, in contrast to our findings, reported no difference between minor complication rates,^2^,^16^ while some studies have findings similar to our study.^17^ In addition, one study that compared the McGrath video laryngoscope with C-MAC video laryngoscope showed no difference in terms of the rates of minor complications, while the number of intubation attempts was higher in the McGrath group. 14 Furthermore, there was no significant difference in the proportional change in hemodynamic parameters, similar to our findings. There was also no significant difference between McGrath video laryngoscope and Macintosh laryngoscope groups in terms of hemodynamic parameters.

In conclusion, the McGrath video laryngoscope produces excellent laryngoscopic views in patients with normal airways, so the practitioner may more easily and comfortably carry out intubation and consider it as the instrument of choice.

## References

[1] Janssens M, Hartstein G (2001). Management of difficult intubation. Eur J Anaesthesiol.

[2] Taylor AM, Peck M, Launcelott S, Hung OR, Law JA, MacQuarrie K (2013). The McGrath®Series 5 videolaryngoscope vs the Macintosh laryngoscope: a randomised, controlled trial in patients with a simulated difficult airway. Anesthesia.

[3] Shippey B, Ray D, McKeown D (2008). Use of the McGrath videolaryngoscope in the management of difficult and failed tracheal intubation. Br J Anaesth.

[4] OLeary AM, Sandison MR, Myneni N, Cirilla DJ, Roberts KW, Deane GD (2008). Preliminary evaluation of a novel videolaryngoscope, the McGrath series 5, in the management of difficult and challenging endotracheal intubation. J Clin Anesth.

[5] Shippey B, Ray D, McKeown D (2007). Case series: the McGrath videolaryngoscope –an initial clinical evaluation. Canadian J Anesth.

[6] Noppens RR, Mobus S, Heid F, Schmidtmann I, Werner C, Piepho T (2010). Evaluation of the McGrath Series 5 videolaryngoscope after failed direct l aryngoscopy. Anesthesia.

[7] Hughes CG, Mathews L, Easdown J, Pandharipande PP (2010). The McGrath video laryngoscope in unstable cervical spine surgery: a case series. J Clin Anesth.

[8] Choi GS, Lee EH, Lim CS, Yoon SH (2011). A comparative study on the usefulness of the Glidescope or Macintosh laryngoscope when intubating normal airways. Korean J Anesthesiol.

[9] Lim TJ, Lim Y, Liu EH (2005). Evaluation of ease of intubation with the GlideScope or Macintosh laryngoscope by anaesthetists in simulated easy and difficult laryngoscopy. Anesthesia.

[10] McElwain J, Malik MA, Harte BH, Flynn NM, Laffey JG (2010). Comparison of the C-MAC videolaryngoscope with the Macintosh, Glidescope, and Airtraq laryngoscopes in easy and difficult laryngoscopy scenarios in manikins. Anesthesia.

[11] Kim W, Choi HJ, Lim T, Kang BS (2014). Can the new McGrath laryngoscope rival the GlideScope Ranger portable video laryngoscope? A randomized manikin study. Am J Emerg Med.

[12] Burdett E, Ross-Anderson DJ, Makepeace J, Bassett PA, Clarke SG, Mitchell V (2011). Randomized controlled trial of the A.P. Advance, McGrath, and Macintosh laryngoscopes in normal and difficult intubation scenarios: a manikin study. Br J Anaesth.

[13] Ruetzler K, Imach S, Weiss M, Haas T, Schmidt AR (2015). Comparison of five video laryngoscopes and conventional direct laryngoscopy: Investigations on simple and simulated difficult airways on the intubation trainer. Anaesthesist.

[14] Ng I, Hill AL, Williams DL, Lee K, Segal R (2012). Randomized controlled trial comparing the McGrath videolaryngoscope with the C-MAC videolaryngoscope in intubating adult patients with potential difficult airways. Br J Anaesth.

[15] Ng I, Sim XL, Williams D, Segal R (2011). A randomised controlled trial comparing the McGrath(®) videolaryngoscope with the straight blade laryngoscope when used in adult patients with potential difficult airways. Anesthesia.

[16] Walker L, Brampton W, Halai M, Hoy C, Lee E, Scott I (2009). Randomized controlled trial of intubation with the McGrath Series 5 videolaryngoscope by inexperienced anaesthetists. Br J Anaesth.

[17] Ray DC, Billington C, Kearns PK, Kirkbride R, Mackintosh K, Reeve CS (2009). A comparison of McGrath and Macintosh laryngoscopes in novice users: a manikin study. Anesthesia.

[18] Choi JW, Kim JA, Jung HJ, Kim WH (2016). Tracheal Intubation with a McGrath®Series 5 Video Laryngoscope by Novice Personnel in a Cervical-immobilized Manikin. J Emerg Med.

